# Histopathologic Features of Immune-related Adverse Events in the Gastrointestinal Tract: A Case of Severe Acute Respiratory Syndrome Coronavirus 2 and Cytomegalovirus Infection in a Patient with Lung Squamous Cell Carcinoma Receiving Immune Checkpoint Inhibitors

**DOI:** 10.14789/jmj.JMJ22-0004-CR

**Published:** 2022-07-14

**Authors:** AKANE HASHIZUME, HIROSHI IZUMI, SHIGEKI TOMITA, TARO OSADA, SHINICHI SASAKI, TAKASHI YAO

**Affiliations:** 1 Department of Pathology, Juntendo University Urayasu Hospital, Chiba, Japan; 1 Department of Pathology, Juntendo University Urayasu Hospital, Chiba, Japan; 2 Department of Gastroenterology, Juntendo University Urayasu Hospital, Chiba, Japan; 2 Department of Gastroenterology, Juntendo University Urayasu Hospital, Chiba, Japan; 3 Department of Internal Medicine and Respiratory, Juntendo University Urayasu Hospital, Chiba, Japan; 3 Department of Internal Medicine and Respiratory, Juntendo University Urayasu Hospital, Chiba, Japan; 4 Department of Human Pathology, Juntendo University Faculty of Medicine, Tokyo, Japan; 4 Department of Human Pathology, Juntendo University Faculty of Medicine, Tokyo, Japan

**Keywords:** immunotherapy, adverse events, colitis, gastritis, cytomegalovirus

## Abstract

In this article, we report the case of a patient with unresectable stage III squamous cell lung carcinoma who developed immune-related adverse events in the gastrointestinal tract following the administration of immune checkpoint inhibitors. The patient developed severe acute respiratory syndrome coronavirus 2 pneumonia and cytomegalovirus gastritis during immunosuppressive therapy for an immune-related adverse event. Cytomegalovirus infection was managed with the administration of ganciclovir.

## Introduction

Immune checkpoint inhibitors are commonly used for the treatment of advanced-stage malignancies. Despite their chance to achieve long-term efficacy, they may induce immune-related adverse events (irAEs). The main irAEs include endocrinopathies, hepatitis, interstitial pneumonia, skin lesions, mucosal inflammation, diarrhea, and colitis. Although most irAEs with severe toxicity are managed with immunosuppressive therapies, occasionally, they can induce infectious disease (e.g., cytomegalovirus infection). This report presents the case of a patient with lung squamous cell carcinoma (SCC) who developed gastrointestinal irAEs, as well as subsequent severe acute respiratory syndrome coronavirus 2 (SARS-CoV-2) and cytomegalovirus infection.

## Case report

**Figure 1:**
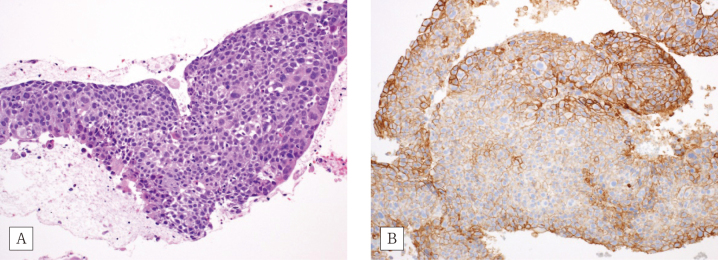
Transbronchial biopsy of squamous cell carcinoma A) Hematoxylin and eosin staining (× 20). B) Immunohistochemical staining of PD-L1 (× 20). PD-L1, programmed cell death 1 ligand 1

**Figure 2:**
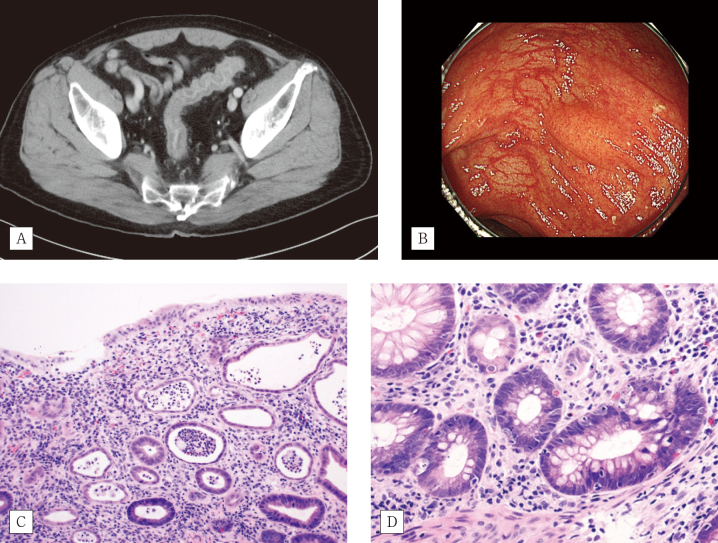
Computed tomography, endoscopy, and histopathological findings of the colon A) Abdominal computed tomography shows bowel wall thickening. B) Mucosal erythema. C) Rectal mucosa with abundant inflammatory cells and atrophic crypt abscess (× 20). D) Prominent crypt epithelial apoptosis (× 40).

A man in his seventies was referred to our hospital following the detection of a nodule in the right lung during a routine health examination. Transbronchial biopsy revealed the presence of SCC. (Figure 1A). The examination for epidermal growth factor receptor (EGFR) mutation, anaplastic lymphoma kinase (ALK) translocation, and ROS1 proto-oncogene receptor tyrosine kinase (ROS1) mutations yielded negative results. The tumor proportion score of programmed cell death 1 ligand 1 (PD-L1) was 95% (Figure 1B). The patient was finally diagnosed with stage IIIA lung SCC, and received concurrent radiation chemotherapy (i.e., carboplatin + paclitaxel + radiation with 60 Gy in 30 fractions). This treatment was followed by maintenance therapy with durvalumab at 2-week intervals. Six months after completion of first-line chemoradiotherapy, he received additional chemotherapy (i.e., nanoparticle albumin-bound paclitaxel + carboplatin + pembrolizumab) for lymph node recurrence. He reported severe diarrhea 10 days after the administration. Computed tomography imaging revealed bowel wall thickening (Figure 2A). He was diagnosed with grade 3 (Common Terminology Criteria for Adverse Events Version 5.0: CTCAE) immune-related colitis and treated with high-dose prednisolone (beginning with 2mg/kg/day and tapering to lower dosage gradually) and infliximab (5mg/kg). Following a limited improvement in abdominal symptoms, he underwent lower endoscopy examination. Colonoscopic findings showed inflammation of the entire colon with a reddish, oedematous mucosa (Figure 2B). Histologically, mixed inflammatory infiltrates with crypt abscesses were observed (Figure 2C). Moreover, increased apoptosis of crypt epithelial cells was observed (Figure 2D). Oral prednisolone (10 mg/day as a maintenance dose) was administered for the treatment of immune checkpoint inhibitor- induced colitis.

**Figure 3:**
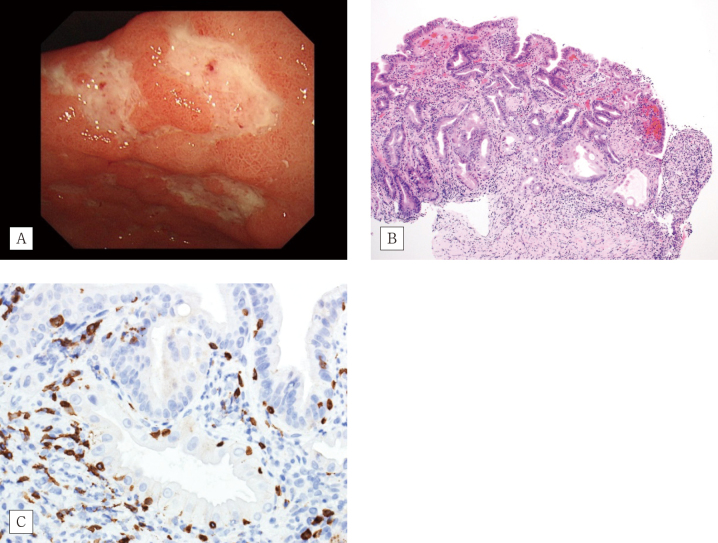
Endoscopy and histopathological findings of the stomach A) Multiple ulcers. B) Gastritis with erosion (× 40). C) CD8 immunohistological staining highlights the lymphocytes (× 40).

**Figure 4:**
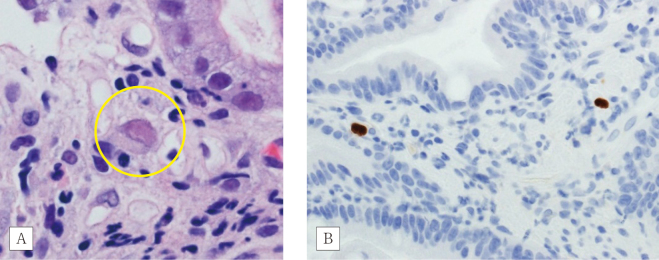
Atypical mesenchymal cells in gastric mucosa A) Intranuclear inclusion body within the circle (× 60). B) Immunohistochemical staining with anti-cytomegalovirus antibody (× 40).

The patient was infected with SARS-CoV-2 during radiation therapy for lymph node metastasis at eight-months after colitis onset. For the treatment of SARS-CoV-2 pneumonia, the patient received 20 mg/day prednisolone and remdesivir. Following recovery from SARS-CoV-2, the dose of prednisolone was tapered to 10 mg/day. He complained of abdominal pain after meals, and underwent endoscopy examination that revealed multiple gastric ulcers (Figure 3A). Histologically, a heavy inflammatory cell infiltrate throughout the mucosa was observed (Figure 3B). The presence of intraepithelial CD8-positive lymphocytes (Figure 3C) suggested an irAE. Atypical mesenchymal cells with an intranuclear inclusion body were found (Figure 4A). Immunohistochemical analysis for cytomegalovirus infection was positive (Figure 4B) and blood examination revealed C7-HRP positivity; hence, we the patient was diagnosed with cytomegalovirus infection. The administration of ganciclovir was effective against cytomegalovirus gastritis.

Written informed consent was obtained from the patient.

## Discussion

Immune checkpoint inhibitors are monoclonal antibodies that block inhibitors of T-cell activation and may cause autoimmune manifestations. The incidence of colitis in patients treated with anti-programmed cell death 1/PD-L1 (anti-PD-1/PD-L1) therapy is <5%^1)^. Any grade irAEs have been reported to occur 32.9% of lung cancer patients treated with pembrolizumab^2)^. In this case, time on pembrolizmab prior to diarrhea was 10days; shorter than median time from PD-1 inhibitor initiation to irAE onset was 3months^3)^. The cause might be the first-line durvalumab.The endoscopic findings of intestinal irAE resemble those of ulcerative colitis^4)^. Inflammatory changes in the entire colon, as noted in the present case, can be observed in patients with inflammatory bowel disease or infectious disease. The morphological changes associated with intestinal irAE are classified into four categories, namely active colitis with apoptosis, lymphocytic colitis, acute self-limiting colitis, and collagenous colitis^5, 6)^^)^. The histopathologic differential diagnoses of intestinal irAE include inflammatory bowel disease, infectious disease, and other therapeutic effects^4, 5)^. Intraepithelial CD8-positive lymphocytosis is a key component in the pathogenesis of irAEs^7)^.

The development of an irAE in immunosuppressed patients is associated with cytomegalovirus infection. Although there are some theories^8^^-^^10)^, the risk of SARS-CoV-2 infection in patients receiving immune checkpoint inhibitors is currently unclear. A study demonstrated that the use of corticosteroids and/or anti-TNF drugs was a major risk factor for the development of infection among patients with melanoma who received immune checkpoint inhibitors^11)^. In this case, it appears that the immunosuppressive agents contributed to SARS-CoV-2 and cytomegalovirus infection. Rectal biopsy using immunohistochemistry, performed at the time of discontinuation of prednisolone or infliximab, was negative for cytomegalovirus. Cytomegalovirus infection should be considered in cases in which a patient with an irAE develops resistance to immunosuppressive therapy. Eroded or cytomegalovirus-infected mucosa sometimes revealed atypical mesenchymal cells which should be distinct from malignancy. The distinction between gastrointestinal irAE and infection is important, because the treatment modalities for these conditions differ considerably. Sufficient clinical information is warranted for accurate pathological diagnosis.

As shown in this report, gastric irAE and cytomegalovirus infection can occur simultaneously during the treatment of colonic irAE. Hence, we should take notice of complication of irAEs and virus infection.

## Funding

The authors received no financial support for the research.

## Author contributions

AH, HI, ST, and TY performed the histological evaluation; TO, SS performed data analysis and interpretation; AH, ST wrote the manuscript; and all authors approved the final manuscript.

## Conflicts of interest statement

The authors declare that they have no conflicts of interest.

## References

[1] Johnson DB, Chandra S, Sosman JA: Immune Checkpoint Inhibitor Toxicity in 2018. JAMA, 2018; 320: 1702-1703. 30286224 10.1001/jama.2018.13995

[2] Cortellini A, Friedlaender A, Banna GL, et al: Immune-related Adverse Events of Pembrolizumab in a Large Real-world Cohort of Patients With NSCLC With a PD-L1 Expression ≥ 50% and Their Relationship With Clinical Outcomes. Clin Lung Cancer, 2020; 21: 498-508. 32680806 10.1016/j.cllc.2020.06.010

[3] Gonzalez RS, Salaria SN, Bohannon CD, Huber AR, Feely MM, Shi C. PD-1 inhibitor gastroenterocolitis: case series and appraisal of ‘immunomodulatory gastroenterocolitis'. Histopathology, 2017; 70: 558-567. 28000302 10.1111/his.13118

[4] Yamauchi R, Araki T, Mitsuyama K, et al: The characteristics of nivolumab-induced colitis: an evaluation of three cases and a literature review. BMC Gastroenterology, 2018; 18: 135. 30170560 10.1186/s12876-018-0864-1 PMC6119262

[5] Chen JH, Pezhouh MK, Lauwers GY, Masia R: Histopathologic Features of Colitis Due to Immunotherapy With Anti-PD-1 Antibodies. Am J Surg Pathol, 2017; 41: 643-654. 28296676 10.1097/PAS.0000000000000829

[6] Zhang ML, Neyaz A, Patil D, Chen J, Dougan M, Deshpande V: Immune-Related Adverse Events in the Gastrointestinal Tract: Diagnostic Utility of Upper Gastrointestinal Biopsies. Histopathology, 2020; 76: 233-243. 31361907 10.1111/his.13963 PMC8386137

[7] Bavi P, Butler M, Serra S, Chetty R: Immune modulator-induced changes in the gastrointestinal tract. Histopathology, 2017; 71: 494-496. 28342232 10.1111/his.13224

[8] Robilotti EV, Babady NE, Mead PA, et al: Determinants of COVID-19 disease severity in patients with cancer. Nat Med, 2020; 26: 1218-1223. 32581323 10.1038/s41591-020-0979-0 PMC7785283

[9] Garassino MC, Whisenant JG, Huang LC, et al: COVID-19 in patients with thoracic malignancies (TERAVOLT): first results of an international, registry-based, cohort study. Lancet Oncol, 2020; 21: 914-922. 32539942 10.1016/S1470-2045(20)30314-4 PMC7292610

[10] Luo J, Rizvi H, Preeshagul IR, et al: COVID-19 in patients with lung cancer. Ann Oncol, 2020; 31: 1386-1396. 32561401 10.1016/j.annonc.2020.06.007 PMC7297689

[11] Castillo MD, Romero FA, Argüello E, Kyi C, Postow MA, Redelman-Sidi G: The Spectrum of Serious Infections Among Patients Receiving Immune Checkpoint Blockade for the Treatment of Melanoma. Clin Infect Dis, 2016; 63: 1490-1493. 27501841 10.1093/cid/ciw539 PMC5106605

